# Systems approach to assessing and improving local human research Institutional Review Board performance

**DOI:** 10.1017/cts.2018.24

**Published:** 2018-08-08

**Authors:** John Fontanesi, Anthony Magit, Jennifer J. Ford, Han Nguyen, Gary S. Firestein

**Affiliations:** 1 University of California at San Diego, San Diego, CA, USA; 2 University of California Biomedical Research Acceleration, Integration & Development (UC BRAID), San Francisco, CA, USA

**Keywords:** Common metrics, Institutional Review Board, best practices, clinical trials, quality improvement

## Abstract

**Objective:**

To quantifying the interdependency within the regulatory environment governing human subject research, including Institutional Review Boards (IRBs), federally mandated Medicare coverage analysis and contract negotiations.

**Methods:**

Over 8000 IRB, coverage analysis and contract applications initiated between 2013 and 2016 were analyzed using traditional and machine learning analytics for a quality improvement effort to improve the time required to authorize the start of human research studies.

**Results:**

Staffing ratios, study characteristics such as the number of arms, source of funding and number and type of ancillary reviews significantly influenced the timelines. Using key variables, a predictive algorithm identified outliers for a workflow distinct from the standard process. Improved communication between regulatory units, integration of common functions, and education outreach improved the regulatory approval process.

**Conclusions:**

Understanding and improving the interdependencies between IRB, coverage analysis and contract negotiation offices requires a systems approach and might benefit from predictive analytics.

## Introduction

A central goal of the Clinical and Translational Science Awards (CTSA) initiative is to foster data-driven improvements in medical research infrastructure that “…increase the quality and efficiency of translational research, particularly of multisite trials.” [1] Local Institutional Review Boards (IRBs) have garnered much of this focus in numerous commission, federal agencies, and publications [2–11]. For example, a recent publication explored IRB performance across 5 University of California Schools of Medicine and proposed a model in which the interaction between operational conditions, study characteristics and proposal characteristics determines how quickly proposals are likely to be approved [11]. The multitude of studies have led to reform proposals have included centralizing IRBs [12], increasing federal oversight, credentialing, greater training [10, 13], and increasing resources for personnel and technology [14]. Furthermore, the time required to acquire IRB approval is now a core “common metric” for all CTSA-funded institutions [15].

However, local IRB’s do not operate in isolation but rather function within a larger regulatory environment (see supplementary Figure A) [11, 16–19]. For example, an IRB-approved informed consent form (ICF) must inform a subject about their potential financial liability. That financial responsibility is negotiated between the contracting unit, the investigator and the sponsoring agency. The terms of that contract are, in turn, reviewed by the institution’s Medicare billing coverage analysis unit to assure compliance with the Centers for Medicare and Medicaid Services regulations. All 3 units have different regulatory oversight for the same section of the consent form and must coordinate their respective language before the consent can be released and a study initiated.

The University of California at San Diego (UCSD) Altman Clinical and Translational Research Institute has prioritized improving the performance of the human research regulatory environment. This effort led to the creation of the Workflow, Outcomes and Quality Improvement Office, which collects and analyzes operational data, conducts data-driven quality improvement (QI) studies and modifies the workflows of regulatory offices. The present study is a novel approach through the systems analysis to explore the relationship between the various offices and identifies actionable data that can improve performance.

## Methods

### Data Collection and Analysis

This process adhered to the International Council on Systems Engineering Standards (INCOSE) QI [20]. Systems engineering is based on the fundamental principle that the world is composed of inter-related systems such that improvement in any one system requires understanding both the nature of that component and the environment within which it exists. INCOSE QI standards are highly detailed, incremental, iterative and flow through a series of prescribed steps from project planning, project assessment, project control, decision making, data analysis, through alternative strategy risk and opportunity assessment to data analysis/modeling, exploring alternatives to the “*AS-IS*” state, pilot testing and re-assessment.

Each stage begins with a declaration of the “customer’s” requirements, in our case—to understand and improve the regulatory environment for clinical research at UCSD. This methods and analysis are an example of how INCOSE QI methods can be used to improve the regulatory environment affecting human subject research with a specific focus on how data analysis can identify the actionable interdependencies between different elements of the regulatory environment.

### Parameters Selected for Study

In the data collection process, we identified 18 entities at UCSD with direct influence on the approval of clinical research studies (see supplementary Figure A for an organizational flow chart). Based on interviews with the unit directors, we selected the 2 units perceived to have to most frequent interactions with UCSD IRB: Office of Coverage Analysis and Office of Contract Analysis (see supplementary Figure B for a high-level flow chart).

We then interviewed and directly observed researchers applying for regulatory approval, analyst reviewing these applications and faculty IRB committee members reviewing new proposals.

Based on this preliminary work and review of the literature, we then selected data fields from the IRB, Office of Coverage Analysis and Office of Contract Analysis data management systems for analysis, using ISO 9004:2000 for process and productivity measures, ISO/IEC 15504 for technology measures and ISO 2859-1 and ANSI/ASQC Z1.9-1993 for sampling procedures [21, 22]. This amounted to 110 data fields contained in 8200 IRB, 4200 Coverage Analysis and 350 new contracts submitted between 2013 and 2016. We constructed and verified data dictionaries for the data fields followed by data cleaning, verification, and validation using Cross-Industry Standard Process for Data Mining (CRIP-DM) methodology [23].

### Data Analysis

Following data cleaning, verification, and validation procedures, the next step in analyzing interdependencies is to explore the distributional characteristics of the data elements. This is an often overlooked but critical QI step or, when done, non-normally distributed data are “normalized” by removing outliers. INCOSE QI uses the distribution family to select appropriate statistical techniques and explores “outliers” as “special cause” or “assignable cause” of undesirable performance variation requiring separate analysis and interventions [24]. As part of our QI effort, we developed predictive models of outliers using 2013–2015 data to “predict” 2016 performance data to assess the stability of the factors creating “outliers” to help determine the feasibility of, first identifying new proposals likely to become outliers and, then, providing a more interventional path to help the investigators address likely challenges in acquiring reserving regulatory approval in a timely manner.

### Statistical Methods

Following INCOSE methodology, non-normally distributed data are reported using quintile regression, such as Kruskal-Wallis ANOVA, and predictive analytics [25, 26] with the direction and dispersion of the data best represented as medians, 25th and 75th percentiles [27]. “Outliers” were analyzed using K-Means and Tree Clustering data mining techniques. Both data mining techniques can be used when there is a mixture of Gaussian distributions.

## Results

### Time Required for IRB Approval

**Fig. 1:**
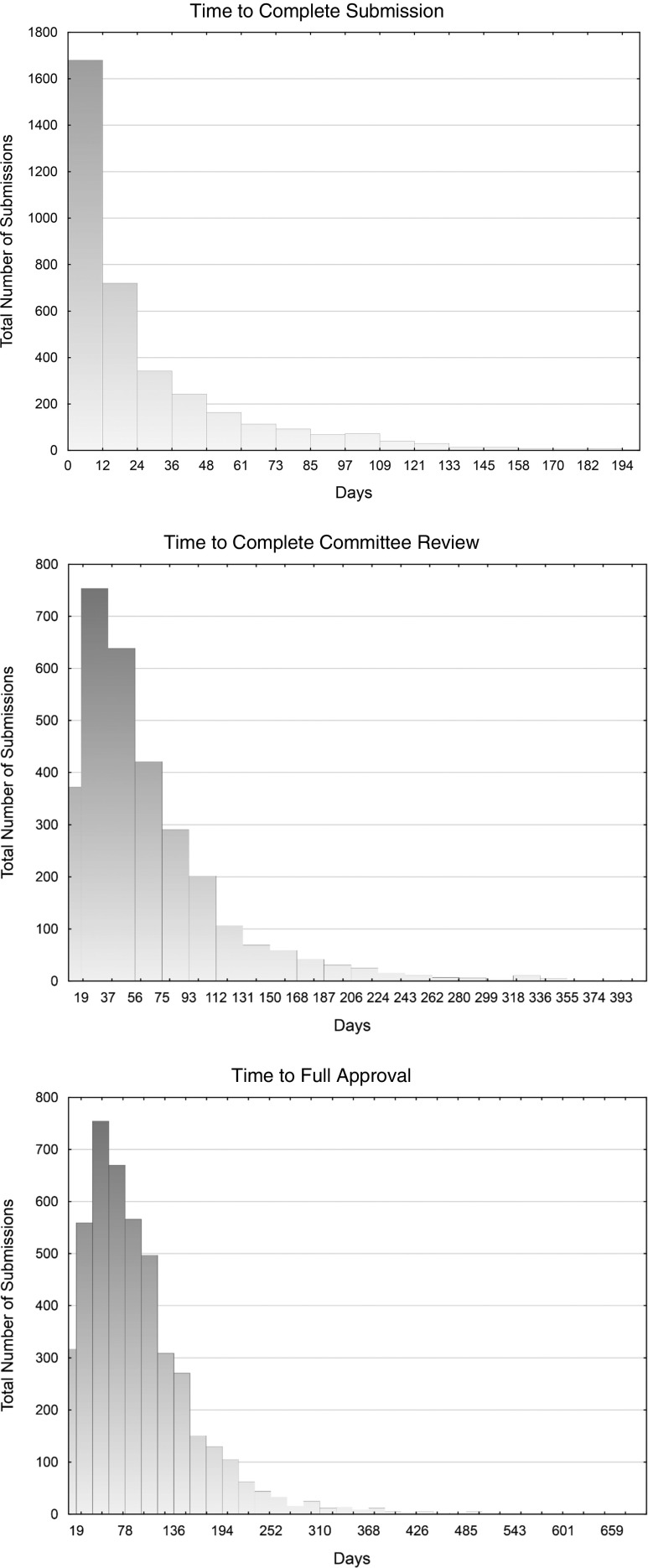
Distribution of protocol time for approval for administrative review, committee review and full approval. Vertical axis shows number of protocols and horizontal axis shows number of days for approval.

In total, 3389 new human subject research protocols, including 618 commercial sponsored studies, submitted to the UCSD IRB Regulatory Environment (IRB environment) between January 2013 and December of 2014 were used for baseline analysis and modeling characteristics affecting time to approval. The first step in the INCOSE methodology was to determine the distribution family, which proved to be a bounded Johnson Distribution with a highly skewed right-sided tail for IRB approval times (Fig. 1). The highly skewed right-sided tail is composed of statistically significant “outliers” identified using Tukey one-sided test (*p=*0.0001). Further analyzing outliers creates an opportunity to identify variations affecting performance that might benefit from a change in the workflow [28–31]. Outliers were independently identified for 2 key phases of the approval process: (1) administrative review by staff; and (2) IRB committee review (see below).

### Contribution of Ancillary Reviews to Delays

**Fig. 2:**
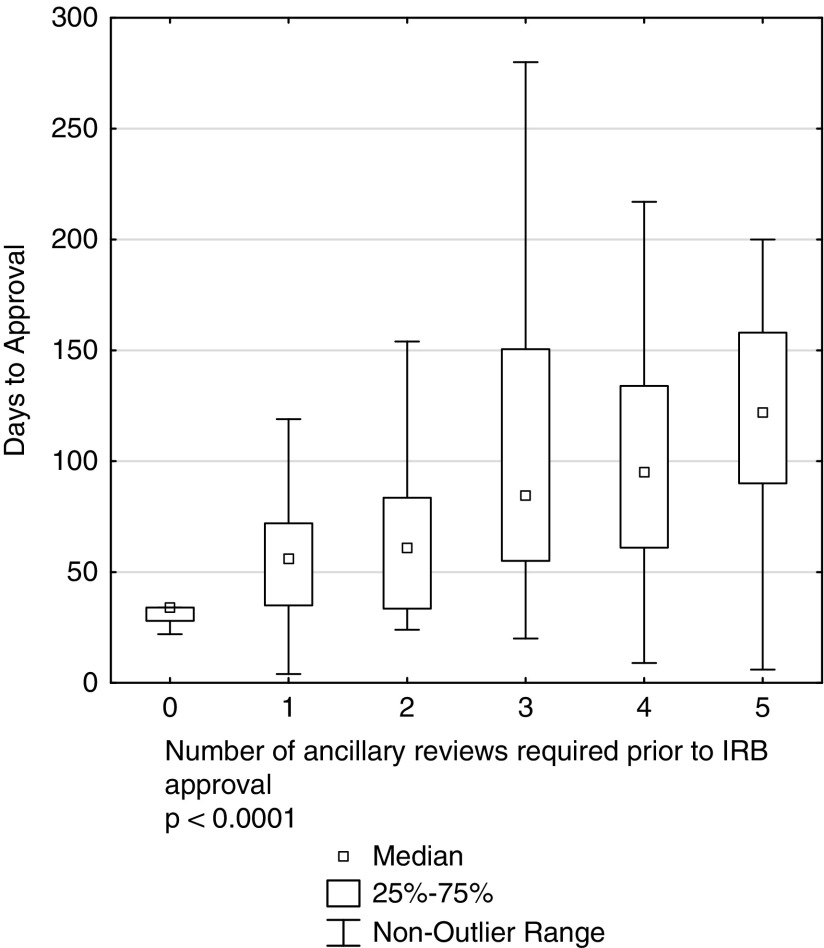
Statistically significant relationship between number of ancillary reviews and time to Institutional Review Boards (IRB) approval.

Both the type and number of ancillary reviews affect the time required to receive IRB regulatory approval (Fig. 2) (*p*<0.0001). Ancillary reviews also added a median of 36 days (interquartile=19–64) post IRB committee approval for release of the consent form. Of interest, UCSD proposals undergo more ancillary reviews than many comparable institutions [11]. For example, UCSD phase III multisite studies had a median of 3 ancillary reviews (interquartile=2–4) whereas data from the previous UC-wide study showed that other UC campuses operating under the same university policy had a median of 0.9 reviews (interquartile=0.2–1.5). The most commonly co-occurring ancillary reviews at UCSD during the initial review period were Radiation Safety Committee (50% of the time) and Scientific Review Committee (35% of the time). The ancillary reviews associated with the longest approval times were the Scientific Review Committee (median=120 days, interquartile=62–147), Biosafety Committee (median=118 days, interquartile=70–148), and Radiation Safety (median=116 days, interquartile=83–147).

### Contribution of Study Type and Study Misclassification

Unexpectedly, non-interventional applications such as anonymized registries, retrospective chart reviews, or observational studies had longer approval times then interventional clinical trials. In exploring where delays occurred, the administrative review had a median duration of 23 days while committee review required a median of 104 days (*p*=0.0018). This counter-intuitive observation is similar to findings from other investigators [29]. Focus groups conducted with researchers suggested that the explanation related to researchers not understanding the criteria and documentation requirements non-interventional studies. This led to requests for expedited reviews or requests for exemptions when full IRB review was required. In addition, 63% of non-interventional studies indicated in the submission documentation that the protocol was a clinical trial, which also created administrative delays.

### Contribution of Staffing Ratios

**Fig. 3:**
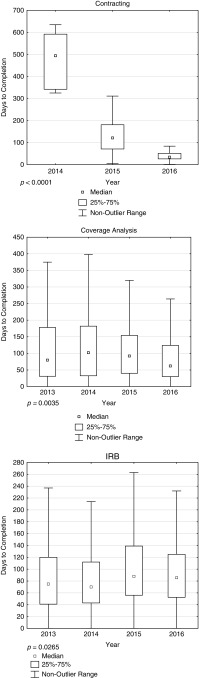
Statistically significant changes in contracting, coverage analysis, and Institutional Review Boards (IRB) approval times. Contracting and coverage analysis performance has improved since 2014. IRB timelines have modestly increased primarily due to static staffing ratios.

Our previous study across the University of California identified IRB staffing ratios were critical determinants of approval times [11]. This finding was confirmed with the larger UCSD data set, where static full-time employee (FTE) staffing over time in the face of increasing number of new applications changed the ratio of new applications per FTE from 69:1 in 2013 to 72:1 in 2016 (see supplementary Table C). The increasing ratio of new protocols to FTEs was accompanied by a rise of median administrative time from 13 days (interquartile=6–34) in 2013 to 25 days (interquartile=15–50) in 2016 (*p*<0.0001). Increased administrative review times meant overall approval times remained essentially static with a median of 75 days (interquartile=42–122) in 2013 to a median of 86 days (Interquartile=53–126) in 2016 (Fig. 3). The increased durations were accompanied by an increase in the number of withdrawn applications, largely due to delays for commercial sponsored studies when deadlines passed. For example, 9 IRB and 23 contracts were withdrawn in 2013. That number increased to 43 IRB and 68 contract withdrawals in 2014 (*p*<0.001, *p*=0.0182, respectively).

### Contribution of Individual Committee Performance

The second operational issue was statistically significant difference between IRB committees. Comparisons were restricted to Phase III multisite commercially sponsored clinical trials to control for study type (see supplementary Figure D). The median time for review and approval for one committee at 53 days (interquartile=25–89) was statistically significantly longer than the other committees (*p*=0.0036). Evaluation of the workflow revealed that the delays were due to duplicate scientific review for these studies even though protocols for commercial sponsored studies cannot be altered and have undergone extensive regulatory review at the U.S. Food and Drug Administration. To resolve this issue, additional training as well as performance metrics of each committee were provided to the committee chairs with the longest timelines. Subsequent follow-up analysis showed that the timelines matched the other committees with identical median times for phase III multisite studies.

### Contribution of Outside Entities and Contract Office Staffing

**Fig. 4:**
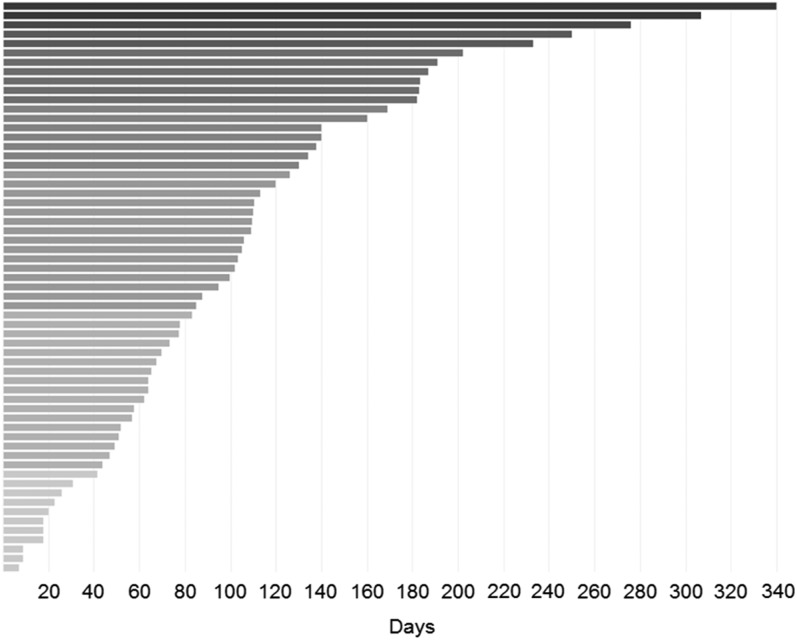
Variance in time for contract execution varies for different contract research organizations (CROs). Each horizontal line represents one CRO for whom at least 4 contracts were negotiated.

Commercial clinical trial sponsors frequently use contract research organizations (CROs) to negotiate contracts and launch a study. Contract delays are translated into delays in IRB ICF release and are typically viewed by the research unit as an “IRB delay.” One of the most striking finding was that individual CROs varied dramatically in terms of timeliness of contracting despite the fact that they all work with the same university contracting office. Fig. 4 shows that there is wide variation in contract execution for various CROs, typically related to responses to queries and contract terms. In addition, Fig. 3 shows how improved staffing and training significantly decreased overall timelines for contracting.

### Contribution of Coverage Analysis

A coverage analysis for protocols that could involve third-party payers, like defined contract language, is required before the ICF is released. Although the early days of centralized coverage analyses created significant delays, improved staffing ratios and training led to a dramatic decrease in the time for completing coverage analyses (Fig. 3, *p*=0.0035). An additional policy change at UCSD delegated authority to the coverage analysis office rather than the research unit to assign a procedure as billable to third parties or the sponsor. This change minimized prolonged negotiations with most research units, which can decline a study if the approved coverage analysis is not acceptable to that unit. An additional finding is the number of study arms within a proposal affects the time required to complete coverage analysis (*p*=0.0006). Each arm requires a separate coverage analysis, which accounts for the longer timelines (see supplementary Figure E).

### Identifying Outliers to Improve Regulatory Performance

**Fig. 5:**
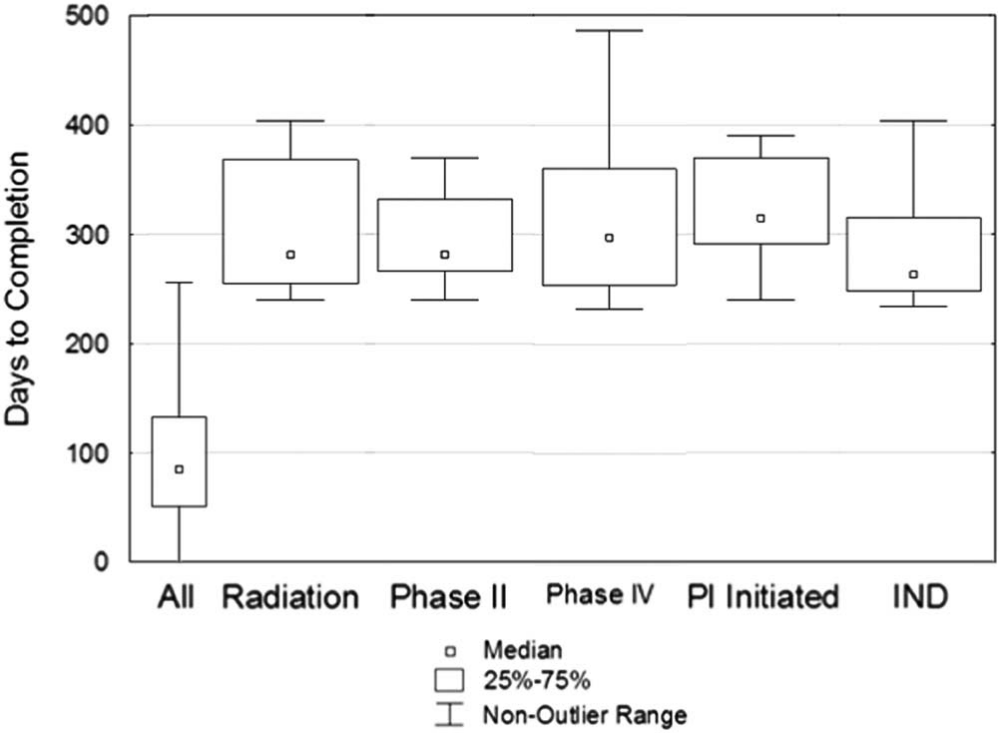
Statistically significant relationship between study characteristics and likelihood of a study being an outlier. Comparison with all studies (All) shows that certain study characteristics can substantially increase the time for completing approval. IND, Investigational New Drug Application: PI, principal investigator.

Analysis of study characteristics for outliers, defined as observations which fall more than 1.5 times the interquartile range, shows some of the key distinguishing features, including principal investigator (PI)-initiated studies, radiation safety review, and testing devices (Fig. 5). We further explored study characteristics associated with the 2 major stages comprising the total approval process: staff review and committee review times. Staff review outliers have a higher percent of scientific reviews, new faculty as the PI, vulnerable populations as research subjects and requests for “reliance” on an external IRB. An example of one “clustering” of study characteristics likely to become an outlier is a PI-initiated study submitted by junior faculty member involving incarcerated juveniles. Another example would be a junior faculty member submitting a cancer study that would include reliance on another institution’s IRB review and approval.

Committee outliers were disproportionately comprised of unfunded studies, PI-initiated studies with protocols requiring radiation, scientific and conflict of interest reviews. An example would be a PI-initiated cancer study in which the investigator had a potential conflict of interest, where treatment of metastatic disease was guided by genomic data and included radiation therapy

### Algorithm to Identify Outliers

**Fig. 6:**
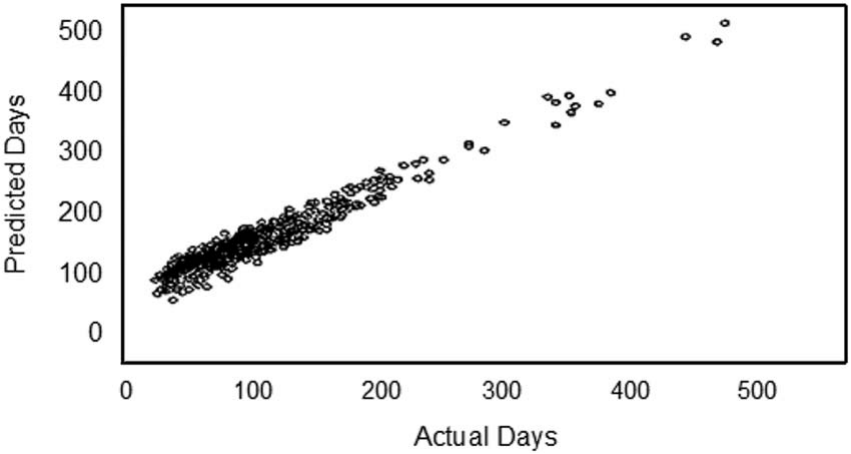
Correlation between predicted and actual times for IRB approval. The algorithm to predict outliers (Table 1) was tested against an independent dataset and showed a significant correlation.

The outlier data were used to develop an algorithm 
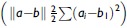
 to identify studies with a high probability of significantly delayed approval. To confirm the approach, we the used K-Means and Tree Clustering to identify subgroupings that were in turn used to create Random Forest predictive algorithms. Table 1 shows the key elements that contribute to outlier status for administrative review, committee review, and total time for approval. The predictive algorithms were then tested against 3828 study protocols submitted to the IRB in 2015–2016. The predictive algorithms identified 88% of the administrative outliers and 93% of the committee outliers (see Fig. 6).

**Table 1:** Random Forest accuracy in predicting 2015–2016 Institutional Review Boards administrative outliers and committee review outliers

	Predictor importance Response: outlier	
	Variable Rank	Importance
*Institutional Review Boards administrative outliers*		
Scientific Review Committee required	100	1.000
Junior faculty	99	0.987
Vulnerable population	97	0.965
Reliance	32	0.315
Medicare billing/coverage analysis required	21	0.208
Biosafety Review required	18	0.177
PI-authored	7	0.0666
	Predicted	
Actual	Yes (%)	No (%)
Yes	88.1	11.90
No	14.75	85.25
Overall accuracy	85.98	85.98
	Predictor importance	
	Variable Rank	Importance
*Committee review outliers*		
Number of ancillary reviews required	100	1.000
Conflict of Interest Review Required	58	0.581
Unfunded	57	0.573
Medicare billing/coverage analysis required	52	0.516
Biosafety Review required	46	0.463
Scientific Review Committee required	16	0.157
Oncology/Hematology	15	0.153
Radiation Safety Review required	12	0.115
PI-authored	2	0.0244
	Predicted	
Actual	Yes (%)	No (%)
Yes	100.00	0.00
No	100.00	0.00
Overall accuracy	93.90	93.90

PI, principal investigator.

### Overall Impact

While it is difficult to ascribe overall improvement to any one initiative, improved education, staffing ratios, and policies together contributed to significant improvement in the performance of the UCSD IRB environment between the 2013–2014 baseline compared with 2015–2016. The impact was particularly evident in contracting and coverage analysis timelines (Fig. 3). However, IRB approval times have modestly increased. This was largely due to the increased in applications per FTE discussed earlier (Contribution of Staffing Ratios section) and has recently been addressed by increasing the staffing for the IRB administrative office.

## Discussion

In this study, we evaluated IRB performance in the context of a larger regulatory environment that not only includes the IRB but also ancillary reviews, clinical trial contracting and research billing coverage analysis. The interactions between the various regulatory units and the researchers trying to navigate them are dynamic and subject to misinterpretation of data. The IRB often bears the ultimate “blame” for delays in the eyes of the investigator even though the committees often are awaiting ancillary reviews, contracts, and coverage analysis simply because the final common pathway for all delays is ICF release.

One important caveat is that “speed” is not always the optimal outcome. In some cases, serious contract issues need to be resolved or the protocol might not incorporate sufficient power or scientific rigor. These situations might require a pause or delay in order to assure compliance or an improved risk:benefit profile. However, operational inefficiencies, such as improper staffing ratios or training, duplicate reviews, or unnecessary ancillary reviews are clearly areas that can be addressed. Collaboration between regulatory units and incorporation of simple solutions like proper staffing, training, project management, and sharing information between regulatory units can overcome some of these barriers.

We have also developed an algorithm for predicting outliers, which can be used to remove those protocols from the normal workflow. Key study characteristics, including multiple ancillary reviews for IRB, multiple study arms for coverage analysis (especially in cancer) and specific CROs for contracting, can be proactively identified. Ultimately separate workflows could be developed for these protocols, which could improve the timelines for other protocols because the outliers will not consume as much resources. Similarly, the causes of administrative delays can be addressed by maintaining adequate staffing and assuring research unit education to improve the initial submission.

This systems engineering approach, in which the processes are broken into individual components, interactions explored and performance drivers identified, involve exploring methods for improving IRB and the entire regulatory process. Unfortunately, centralize control management principles that work in a manufacturing plant are unlikely to work in a human research regulatory environment. Traditional systems are managed to minimize cost while human research should be managed to maximize value. A focus on the efficiency and productivity of the regulatory units is, as noted above, an important value but not at the cost of quality, including patient safety.

Rather than mandate a set of interventions or specific outcomes, our data suggest that a focus on managing complexity by monitoring and influencing the system state might be more beneficial. The organizing principle that promotes understanding and/or appreciation of the role each unit contributes to human research is also required.

A productive approach would be to integrate processes and seek to learn from each protocol and each delay. For example, minor changes in protocols (e.g., same protocol with a different drug in a clinical trial) could be “fast tracked” to assess the safety of the drug rather review the entire protocol as if it had never been seen before by the IRB or other offices. Table 2 provides a matrix for exploring the interdependencies and well as identifying some potential solutions. The challenges outlined will also be true for a centralized IRB.

**Table 2:** Suggested analytic framework for Institutional Review Boards quality improvement efforts

Dimension	Potential contributors	Potential solutions
Staffing workload	∙ Inadequate staffing ∙ Wrong mix ∙ Training ∙ Inadequate infrastructure	∙ Benchmarking ∙ Develop business case ∙ Identify key content knowledge and work ethics ∙ Explore options for supportive technology
Committee workload	∙ Meeting frequency ∙ Volume and types of reviews ∙ Training/understanding ∙ Member knowledge and expertise ∙ Committee dynamics ∙ Infrastructure	∙ Benchmarking ∙ Feedback/policy/ Education ∙ Explore options for supportive technology
Application	∙ Researcher knowledge of current regulatory requirements	∙ Training/concierge service ∙ Supportive templates and use cases
Regulatory environment	∙ Number of other regulatory units	∙ Institutional policy on sequential or parallel processing ∙ Harmonized requirements and application forms ∙ Supportive templates and use cases

## Conclusion

We identified and reviewed issues and delays associated with the regulatory environment, with an emphasis on IRB processes in the context of a larger regulatory universe. Relatively simple steps can have a significant impact on timelines and, ultimately, on researcher satisfaction and patient access to novel therapies. The next steps will entail analyzing the impact of these steps on timelines and quality.

## Supplementary material

For supplementary material accompanying this paper visit https://doi.org/10.1017/cts.2018.24. click here to view supplementary material
