# Supplementary material for: Systems approach to assessing and improving local human research Institutional Review Board performance
Source: J Clin Transl Sci. 2018 Aug 8;2(2):103–9. doi: 10.1017/cts.2018.24 (PMC6799096; doi:10.1017/cts.2018.24)
Supplement: Supplementary file 1 [file S2059866118000249sup001.docx]

Figure A: High-level Workflow of the UCSD Regulatory Environment


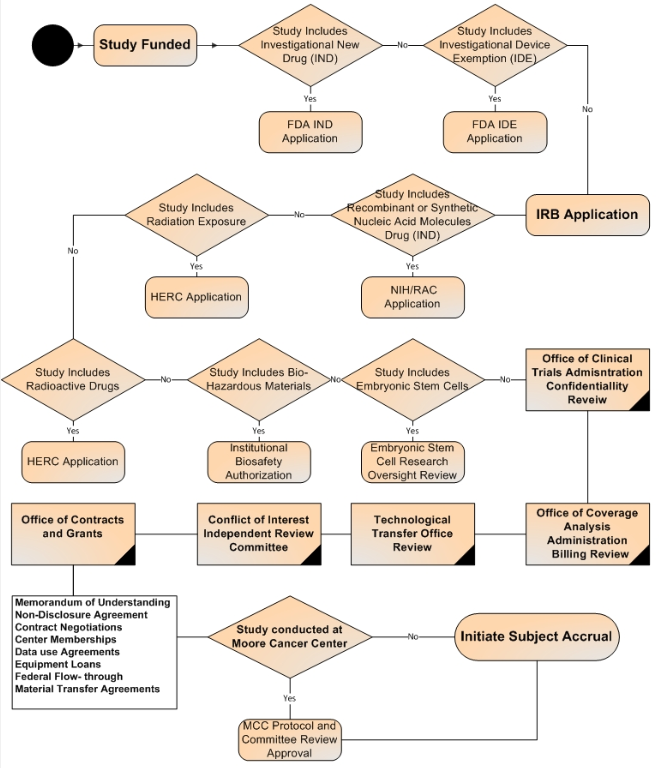


Figure B: Simplified Workflow Travel lanes Diagram of UCSD IRB, Office of Coverage Analysis and Office of Contracts and Grants


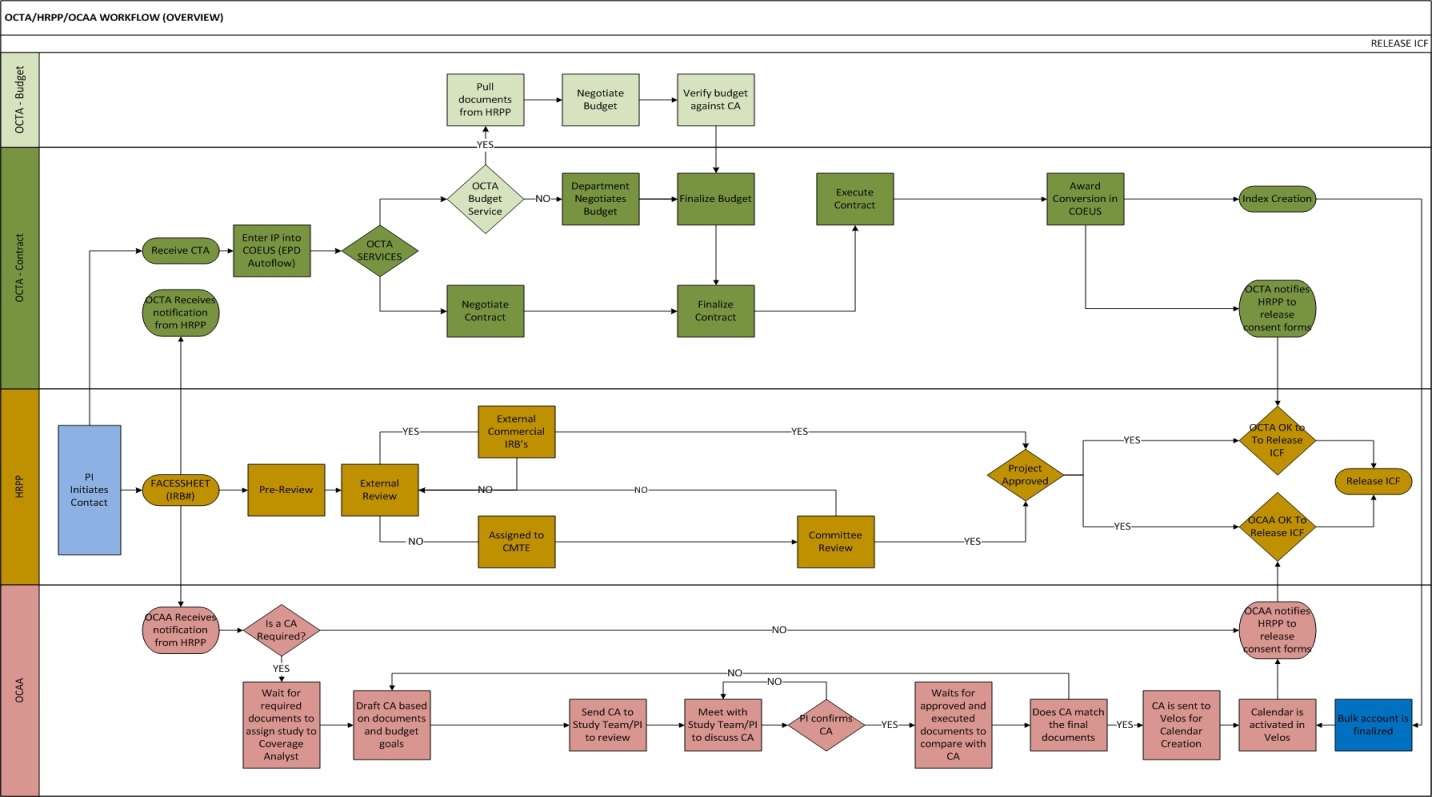


Table C

Table Showing Relationship between Number of Protocols per FTE Analyst and Time Required to receive IRB Approval

| Year | FTE Analysts | Ratio of Protocols to FTE’s | Median Days Administrative Review | Median Days Committee Review | Median Days to Approval (Interquartile) |
| --- | --- | --- | --- | --- | --- |
| 2013 | 17 | 69:1 | 13 | 50 | 75 (42/122) |
| 2014 | 17 | 66:1 | 14 | 40 | 70 (43/112) |
| 2015 | 17 | 70:1 | 19 | 47 | 88 (56/140) |
| 2016 | 17 | 72:1 | 25 | 48 | 86 (53/126) |

Figure D: Statistically Significant Differences in Approval Times Between IRB Committees for Phase III Multi-Site Clinical Trials. Additional training for the outlier committee improved performance and decreased the differences compared with other committees.

Figure E: Statistically Significant Relationship Between Number of Study Arms and Time to Complete Coverage Analysis. Coverage analysis for studies with multiple arms requires substantially more time.
